# Supplementary material for: Side-Chain-Assisted Transition of Conjugated Polymers from a Semiconductor to Conductor and Comparison of Their NO2 Sensing Characteristics
Source: Materials (Basel). 2023 Apr 4;16(7):2877. doi: 10.3390/ma16072877 (PMC10095908; doi:10.3390/ma16072877)
Supplement: Supplementary file 1 [file materials-16-02877-s001.zip › materials-2297962-supplementary.pdf]

Supporting information

Side-Chain-Assisted Transition of Conjugated Polymers from a Semiconductor to Conductor and Comparison of their NO2 Sensing Characteristics

Yejin Ahn, Sooji Hwang, Hyojin Kye, Min Seon Kim, Wi Hyoung Lee, and Bong-Gi Kim *

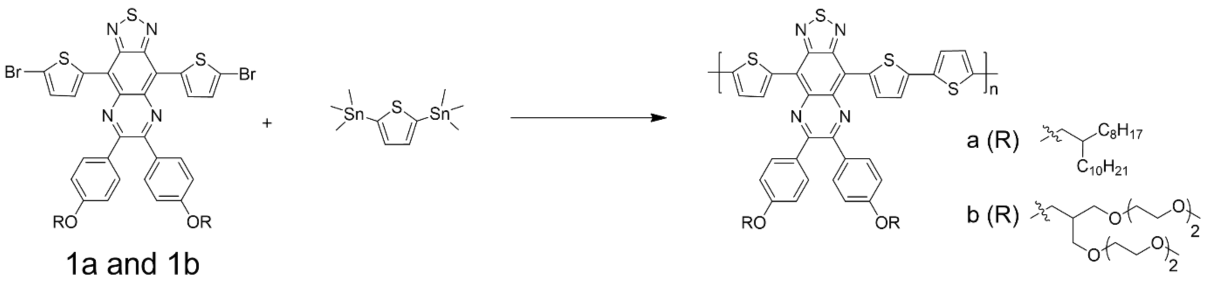

Scheme S1. Detailed chemical structures and polymerization scheme of CPs.

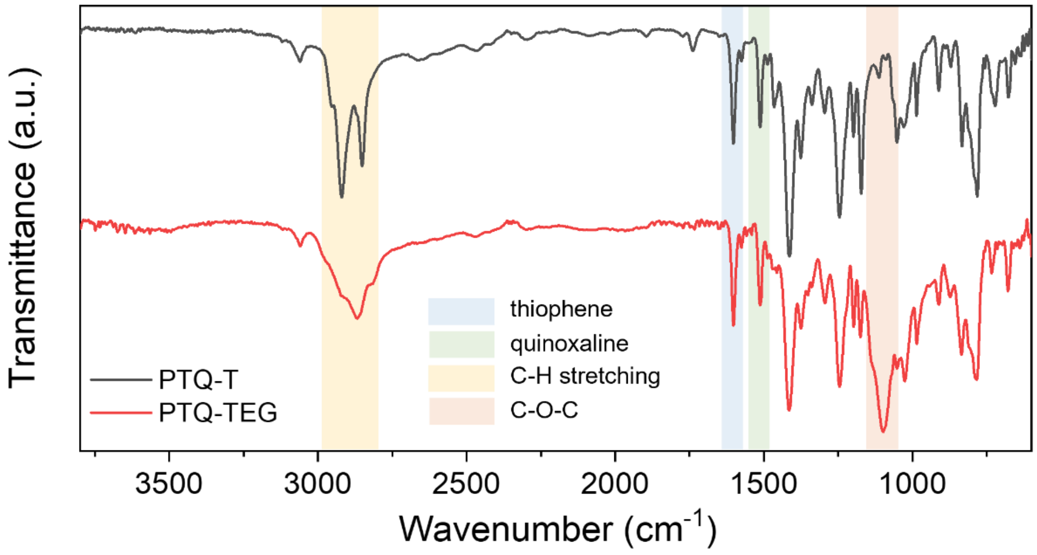

Figure S1. FT-IR absorption spectra (a) **PTQ-T** and (b) **PTQ-TEG**.

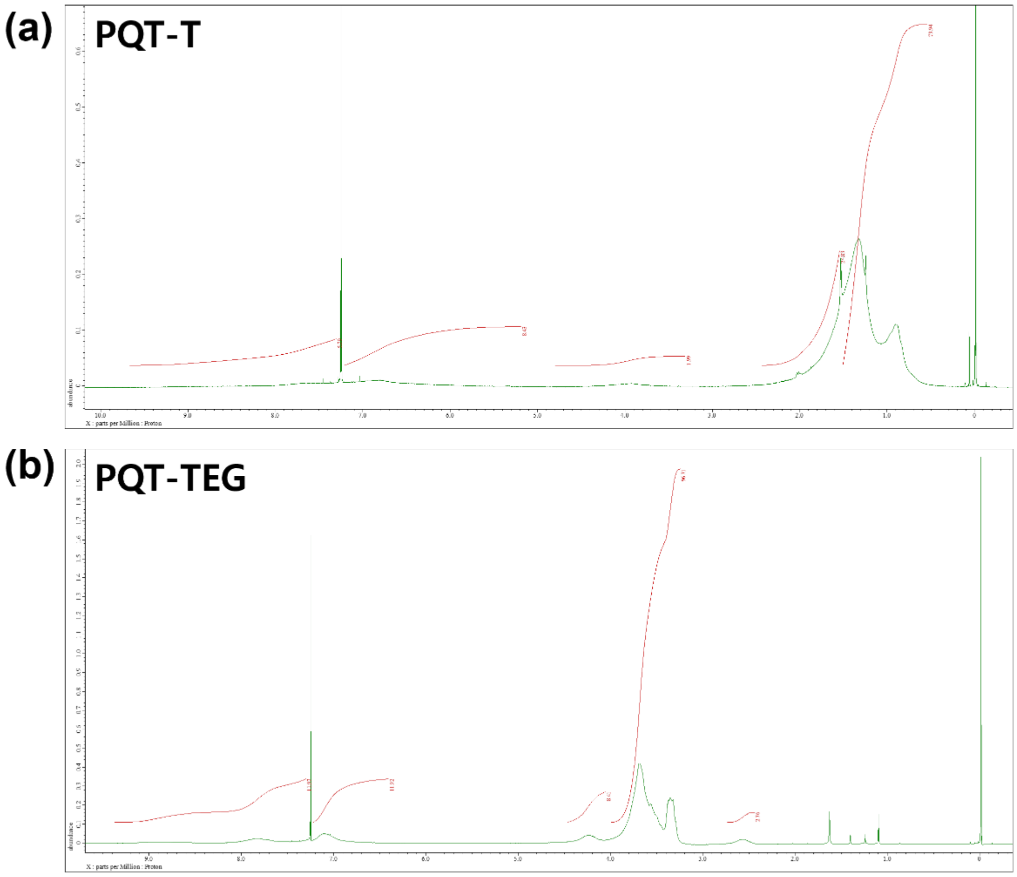

Figure S2. ¹H-NMR spectra of (a) **PTQ-T** and (b) **PTQ-TEG**.

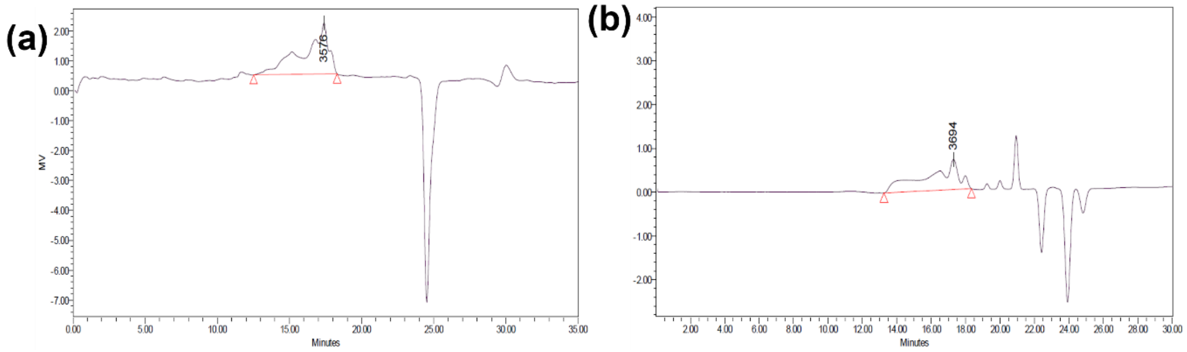

Figure S3. Molecular weight of (a) **PTQ-T** and (b) **PTQ-TEG**, which were determined using gel permeation chromatography with elution of chloroform.

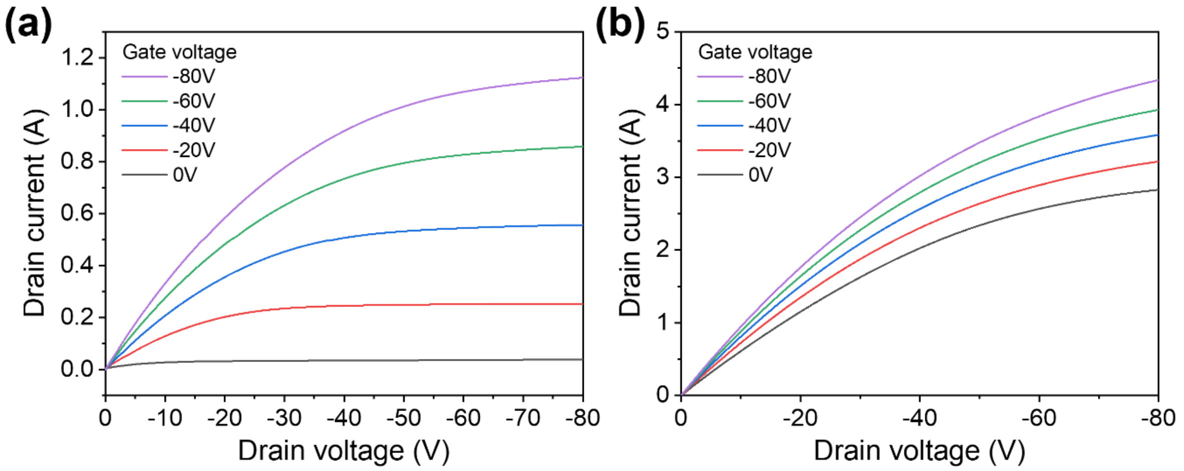

Figure S4. Output curve characteristics of (a) **PTQ-T** and (b) **PTQ-TEG** according to V_G in FET device.

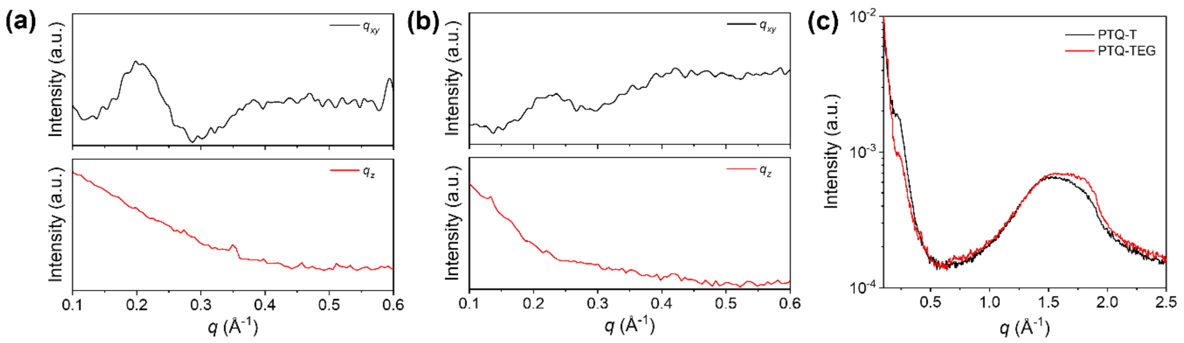

Figure S5. XRD diffraction patterns in the small q region of (a) **PTQ-T** and (b) **PTQ-TEG**, and (c) average XRD diffraction patterns of all direction in both CPs.

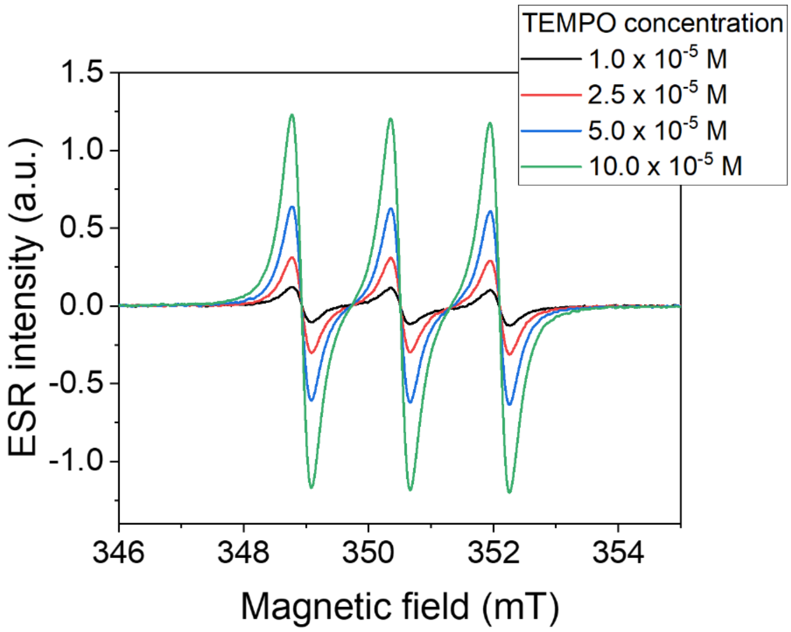

Figure S6. ESR intensity according to TEMPO concentration.

	PTQ-T	PTQ-TEG
Water	85.21° 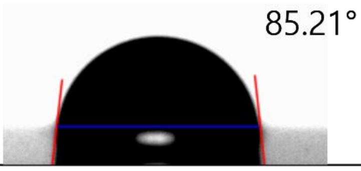	68.06 ° 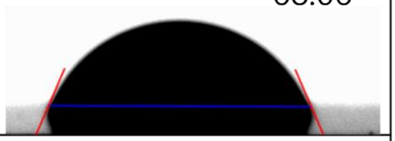
Diiodomethane	41.07° 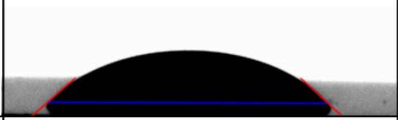	34.07° 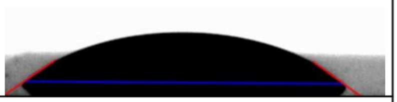
Glycerol	76.32° 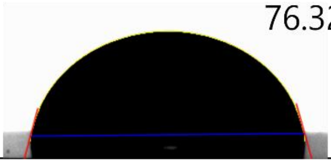	60.62 ° 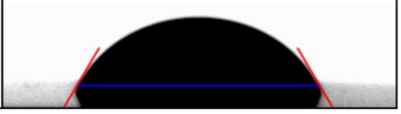

Figure S7. Contact angle of each solvent on the CP films.

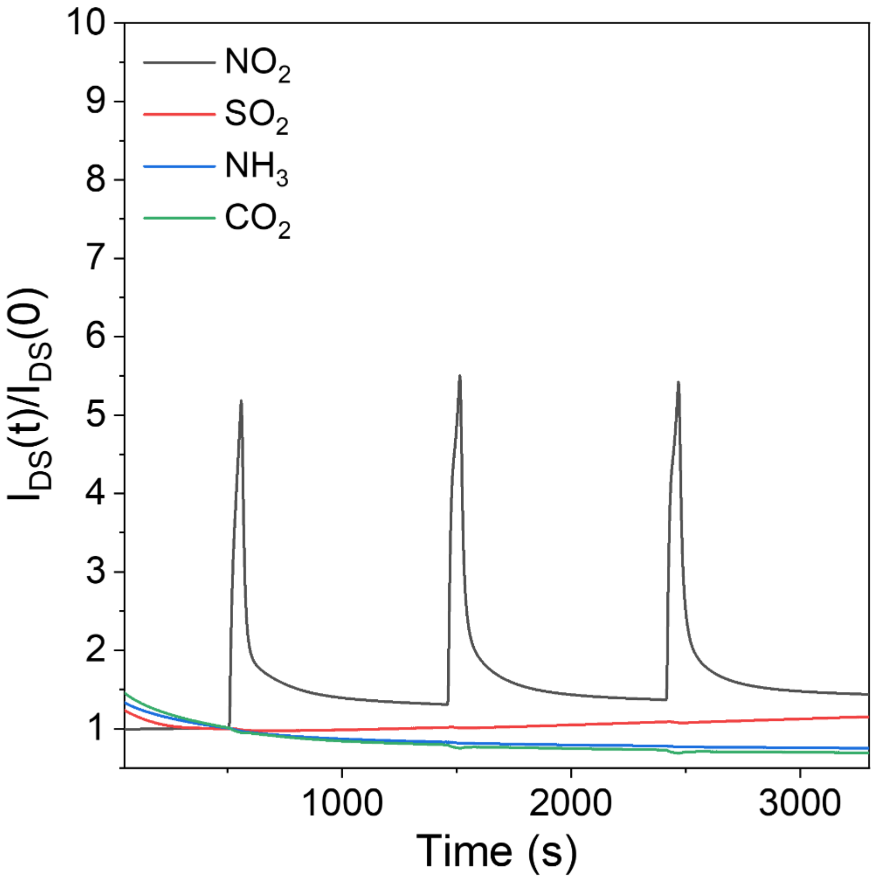

Figure S8. Response pattern of **PTQ-TEG** resistive sensor to common gases.

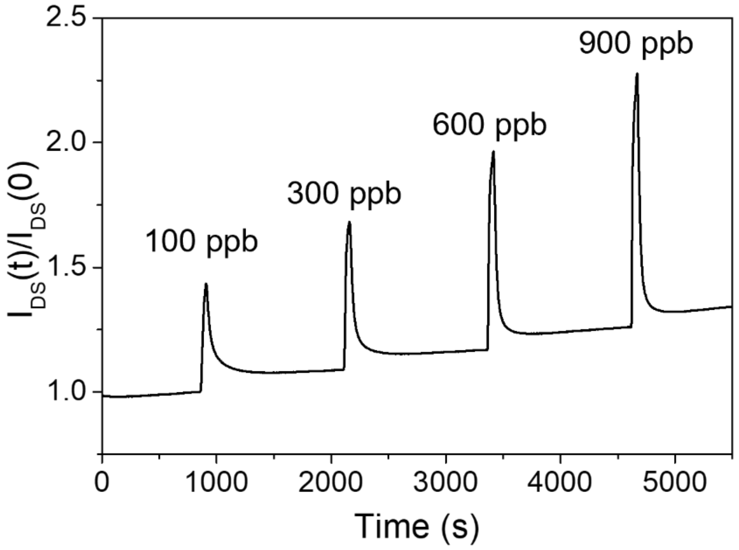

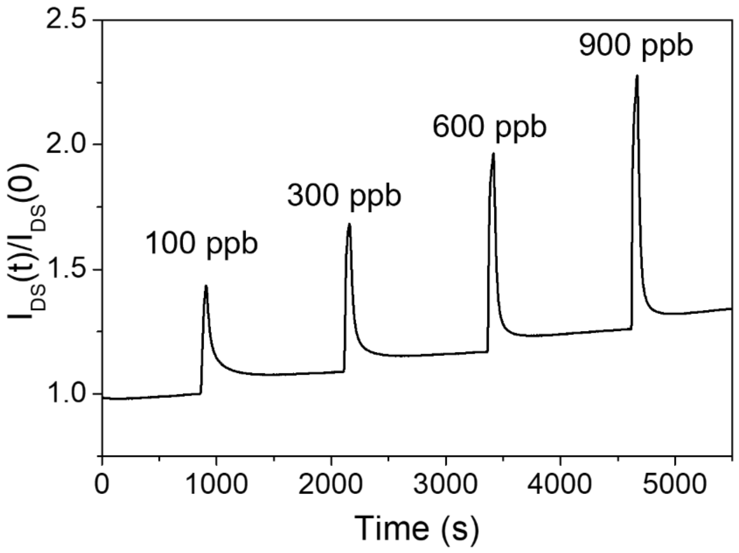

Figure S9. Response pattern of **PTQ-TEG** resistive sensor according to NO_2 concentration.
